# Learning by reading: A review of recent diabetes articles

**DOI:** 10.1111/1753-0407.13272

**Published:** 2022-05-23

**Authors:** Zachary Bloomgarden

**Affiliations:** ^1^ Icahn School of Medicine at Mount Sinai New York USA

The medical literature of research in the development and management of diabetes is endlessly fascinating. The Chinese expression “道山学海” can be translated as, “knowledge is as high as the mountains and as wide as the seas,” expressing this succinctly. A summary of some interesting current articles follows, covering many aspects of the field.

## 1 AUTOIMMUNITY IN DIABETES

A 1‐year pilot study suggested that administration of the antihypertensive calcium channel blocker verapamil 120–360 mg daily vs. placebo to 24 newly diagnosed adults with type 1 diabetes (T1D) preserved C‐peptide with reduction in insulin requirement.^1^ Two‐year follow‐up data have now been reported; those discontinuing verapamil showed reduction in stimulated C‐peptide and increase in insulin requirement, whereas continuation of treatment appeared to maintain the improvement. The neuroendocrine secretory protein chromogranin A (CgA) is a beta‐cell autoantigen with elevated levels in T1D; CgA showed negative correlation C‐peptide secretion in the study, with levels ~50 ng/ml in control persons with T1D, decreasing to ~25 ng/ml with verapamil treatment, and increasing to ~50 on verapamil discontinuation.^2^ Could autoimmunity play a role in the progression to loss of insulin secretory capacity in type 2 diabetes (T2D) as well? A substudy of the Glycemia Reduction Approaches in Diabetes (GRADE) study of persons with T2D having glycosylated hemoglobin (HbA1c) >6.5% on treatment with metformin ≥1 g/day found that 41.3% of 322 participants had islet‐specific T‐cell reactivity, associated with reduction in measures of endogenous insulin secretion; 15.8% of these and 14.3% of those negative for T‐cell reactivity had at least one positive islet autoantibody, but this was not associated with decreased β‐cell function.^3^

## 2 WEIGHT CONTROL APPROACHES AND OUTCOMES

**FIGURE 1:**
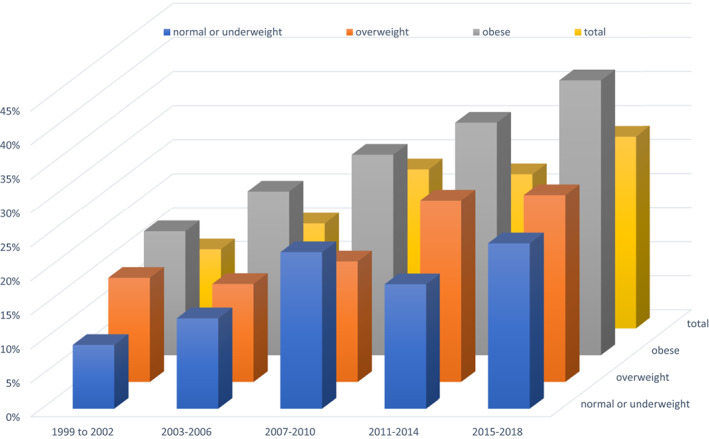
Weighted prediabetes prevalence, US youths 1999 to 2018. Drawn from data in^4^

An analysis of the prevalence of obesity in US youths based on National Health and Nutrition Examination Survey cycles from 1999 through 2018 showed an increase from 19% to 24%, with prediabetes prevalence increasing from 12% of the total population and 18% of obese youths in 1999–2002 to 28% and 40%, respectively, in 2015–2018 (Figure 1).^4^

A metaanalysis of daily step count data from 47 471 participants in 15 studies with 3013 deaths showed that the lowest mortality was at approximately 8000 steps per day, with the appearance of a particularly steep dose–response relationship for participants age 60 and over, for whom relative mortality was 44%, 55%, and 65% lower than that in the lowest activity quartile of participants, with the lowest, second, third, and highest quartiles having median counts of 2841, 5217, 7116, and 10 501 steps per day.^5^ Analyses of the relationship between step count and outcome do not appear to have been carried out among persons with diabetes. With increasing availability of devices measuring step count, heart rate, heart rate variability, caloric expenditure, active time, distance traveled and so on, such epidemiologic studies would offer important tools for encouraging and monitoring healthy lifestyle.

In a study of 2 297 799 persons in the national diabetes registry in England 2003–2018, 38 530 (1.7%) had T2D remission, defined by two HbA1c levels below 6.5% at least 26 months apart without glucose‐lowering medication use. Remission occurred in 3.8% of those with diabetes duration <2 years treated with metformin alone or no medication and with baseline HbA1c <7%, and in 8.3% of those with these characteristics whose body weight also decreased ≥10%, with older age, lesser degrees of socioeconomic deprivation, female sex, and White race further increasing likelihood of remission; although infrequently performed, bariatric surgery was associated with 20.6 % diabetes remission.^6^ A comparison of participants with T2D in four randomized controlled trials, 160 having bariatric surgery vs. 76 having medical/lifestyle therapy, found 38% vs. 3% diabetes remission at 3 years, with a trend to reduction in cardiovascular disease events. Further evidence of benefit of weight loss was seen in 16.7 year follow‐up of the Look AHEAD (Action for Health in Diabetes) study; 934 participants randomized to the intensive lifestyle intervention had ≥10% weight loss during the first year and had a significant 21% reduction in subsequent mortality, not seen in those with <10% weight loss.^7^

In 1‐year follow‐up of 199 persons with T1D treated with a sodium‐glucose cotransporter‐2 inhibitor (SGLT2i), HbA1c decreased from a baseline level of 8.2%–7.7%, with particularly great reduction among those with body mass index (BMI) >27 and among those with baseline HbA1c >8%. Body weight decreased on average by 2.9 kg and insulin requirements decreased by 8.5%. Five and 7 of the patients receiving an SGLT2i developed elevated ketones with and without acidosis, respectively, with a trend to more ketoacidosis among those receiving a higher dose SGLT2i, and genital infections developed in 19% of men and in 26% of women.^8^

## 3 HYPOGLYCEMIA

A well‐recognized association is that of end‐stage liver disease with decreased hepatic capacity for glycogen storage and gluconeogenesis, leading to a pattern of fasting hypo‐ or euglycemia with daytime hyperglycemia. This concept has recently been extended in an analysis of a potential association of non‐alcoholic fatty liver disease (NAFLD) with severe hypoglycemia (SH) in the National Health Insurance Service of South Korea database. Among nearly 2 million persons with T2D followed for a median of 5.2 years, 45 135 had ≥1 episode of SH, with 1.29‐fold increased SH risk in the 10th decile of an NAFLD index based on triglyceride, BMI, waist circumference, and gamma glutamyl transphosphatase level. SH was, as expected, more common with lower BMI but paradoxically was more common with greater waist circumference (perhaps related to the severity of the hepatic disease). Furthermore, the association of SH with NAFLD appeared particularly great in those persons not having chronic kidney disease (CKD) and in those not using insulin,^9^ further suggesting this to be causally related to SH in a fashion different from usually recognized factors.

## 4 MACROVASCULAR DISEASE TREATMENT APPROACHES

In a study of 473 399 adults with T2D registered in the UK Clinical Practice Research Datalink from 2007 to 2018 and followed on average for 6 years, an estimate of the lifetime risk for those over age 45 not having cardiovascular or renal disease was that 9% would develop peripheral arterial disease,19% would have a myocardial infarction, 20% a stroke, 29% would develop heart failure (HF), and 54% would develop CKD.^10^ Another study of the dataset found 18.1 and 13.9 major adverse cardiovascular events (MACE) and HF events per 1000 person‐years of follow‐up among persons with T2D not having prior cardiovascular disease or prior HF, respectively; 2‐3% of persons in the data set used SGLT2i and glucagon‐like peptide 1 receptor agonists (GLP‐1RA), and 0.2‐0.3% used both. Use of SGLT2i, GLP‐1RA, and their combination was associated with 18%, 7%, and 30% lower odds of MACE, and with 51%, 18%, and 43% lower odds of HF events, respectively.^11^

In a propensity score matched analysis of GLP‐1RA use in the Taiwan National Health Insurance Research Database of >4 million persons with T2D from 1998 to 2018, the average compliance rate was 40% overall, but 65% for those with >251 days of GLP‐1RA use; the hazard rate for stroke was 1.03, 0.83, 0.69, and 0.25 for those with 1–59, 60–153, 154–251, and >251 days of GLP‐1RA use, respectively,^12^ confirming randomized controlled trial evidence of benefit of these agents. Another analysis from Taiwan compared persons with T2D initiating glyburide or glipizide, which block cardiac mitochondrial ATP‐sensitive potassium channels, with those initiating gliclazide or glimepiride, not having this effect, from 2007 to 2016; the former agents were associated with 1.2‐fold greater risk of MACE than the latter, with 1.2‐fold greater risk of stroke and 2.6‐fold greater risk of cardiovascular death, with significant 4.7‐fold increase in MACE risk appearing during the first 90 days of use of the agents.^13^

In analysis of outcomes among 5518 participants in the Action to Control Cardiovascular Risk in Diabetes (ACCORD) lipid trial, those randomized to fenofibrate plus simvastatin had an 18% lower likelihood of either HF or cardiovascular mortality than those receiving simvastatin alone, with benefit seen only in the subset randomized to conventional glycemic control, with those receiving intensive glycemic control not showing benefit, and without statistical evidence of mediation by changes in estimated glomerular filtration rate (eGFR), high‐density lipoprotein cholesterol, or triglyceride levels, or by different frequencies of use of thiazolidinediones or insulin.^14^

## 5 NEUROPATHY, RETINOPATHY, AND NEPHROPATHY

Using single nucleotide polymorphism analysis of participants in the UK Biobank (UKB) and the ACCORD trial, the heritability of diabetic kidney disease was 0.29, of microalbuminuria from 0.25 to 0.60, of macroalbuminuria 0.41, and, for more severe degrees of retinopathy, 0.29 for retinal photocoagulation or vitrectomy, and 0.33 for severe vision loss, corroborating the concept that genetic risk comprises an important aspect of diabetic microvascular complications.^15^

In an analysis of US Medicare claims data of 11 638 371 beneficiary‐years for 3 982 684 persons from 2013 to 2019, 78 716 persons, 8.3% of those with diabetes, had at least one diabetic foot ulcer (DFU)episode, an incidence of 4.6 DFU per 100 person‐years among those with diabetes, underscoring the importance of DFU among older persons with diabetes.^16^ Among 699 and 709 participants with T1D in the Diabetes Control and Complications Trial in the intensive vs conventional glycemic control groups for ~6.5 years subsequently followed for 23 years post trial, 86 and 109 developed DFU, respectively, so that prior intensive glycemic control led to a 23% lower likelihood of DFU development, with ulcer risk rates beginning to diverge after the 10th year of follow‐up. HbA1c, greater age, clinical neuropathy, cardiovascular autonomic neuropathy, retinopathy, and albuminuria were associated with risk of development of DFU. In addition, age, BMI, serum triglyceride, macular edema, and smoking were associated with greater risk of lower extremity amputation.^17^

Using data from the 2008–2011 Korean National Health and Nutrition Survey, of 1350 persons with T2D who had retinal evaluation, 20% had diabetic retinopathy (DR), of whom 12.2% had proliferative diabetic retinopathy (PDR) and 5.3% had vision‐threatening retinopathy; perhaps suggesting a therapeutic option, those consuming ≥1 cup of coffee daily had 56% lower likelihood of retinopathy, although the association was significant only among those <65 years of age.^18^ In a metaanalysis of 18 studies of 1464 pregnant women with T1D and 262 with T2D, DR was present in 52.3% of pregnancies and 57.8% around delivery, but in T2D, prevalence was 9.4% in early pregnancy and 17.5% in late pregnancy. PDR was present in 6.1% during early pregnancy and 8.2% around delivery; new DR development occurred in 15%, worsening nonproliferative DR 31%, progression to PDR 6.3%, and worsened PDR occurred in 37%.^19^ A commentary in *JAMA* ophthalmology highlighted the issue of continued vision loss in some patients with DR over long‐term follow‐up, despite optimal laser treatment and administration of antivascular endothelial growth factor therapy and suggested that many of these cases may reflect ganglion cell loss from the less well‐recognized entity of diabetic macular ischemia.^20^

It has been unclear whether nonalbuminuric CKD is associated with risk of poor kidney outcome and death comparable to than that seen in nonalbuminuric persons with diabetes without CKD. In a cohort of 8320 T2D persons followed from 2003 to 2018, nonalbuminuric CKD (urine albumin‐creatinine ratio <30 and eGFR <60) was associated with mortality similar to that with albuminuria without CKD (urine albumin‐creatinine ratio ≥30 and eGFR ≥60), and the likelihood of worsening of CKD also was similar in the two groups.^21^

## 6 PREGNANCY AND DIABETES

In a study of 1 116 779 Danish offspring from 1997 to 2016, among 5298, 1451, and 647 whose fathers were treated with insulin, metformin, and a sulfonylurea risk of birth defects 0.98‐, 1.40‐, and 1.34‐fold greater than the remainder of the population.^22^ Although the increased likelihood was statistically significant for metformin and not for sulfonylureas, the suggested interpretation that paternal exposure to metformin might predispose to birth defects seems unlikely given the similar risk ratio for the two classes of oral hypoglycemic agents, so that if such an association exists it may be one of paternal T2D with birth defects.

In a study of 1414 women with a history of gestational diabetes and 46067 not having such a history, with mean 10.2 year follow‐up, beginning from the 6th year after gestational diabetes, women had a 3.9‐fold increase in subsequent T2D within 6–15 years, 3.5‐fold within 16–25 years, 2‐fold within 26–35 years, and 1.6‐fold after 35 years.^23^
